# Study on the Pumping Performance and Structure Parameters Optimization of High-Speed Small Compound Molecular Pump

**DOI:** 10.3390/mi15060717

**Published:** 2024-05-29

**Authors:** Zhi Chen, Lei Zhang, Zhizuo Li, Zhizhong Zhang, Guojun Zhang, Fenglin Han

**Affiliations:** 1 State Key Laboratory of Precision Manufacturing for Extreme Service Performance, College of Mechanical and Electrical Engineering, Central South University, Changsha 410083, China; 217052@csu.edu.cn (Z.C.); lzz211122@163.com (Z.L.); 2 Guangdong Provincial Key Laboratory of Manufacturing Equipment Digitization, Guangdong HUST Industrial Technology Research Institute, Dongguan 523808, China; 3 AECC Hunan Aviation Powerplant Research Institute, Zhuzhou 410199, China; 18202764498@163.com

**Keywords:** compound molecular pump, pumping performance, thin air environment, aerodynamic model, structure parameters optimization

## Abstract

A molecular pump is the core component of vacuum systems in portable mass spectrometers and other analytical instruments. The forms of the existing molecular pumps mainly are the combinations of vertical bleed and compression channel, which have the shortcomings of heavy mass and large volume, which seriously restricts the application and development of portable mass spectrometers. Aiming at the problems of low strength and insufficient pumping performance under the miniaturization constraints (mass of 1.8 kg; exhaust diameter of 25 mm) of molecular pumps, a compound pump consisting of a horizontal bleed channel and multi-stage spiral compression channel is proposed. The pumping principle of the compound molecular pump is analyzed to obtain its preliminary structural size parameters. The test particle Monte Carlo method is presented for establishing an aerodynamic model for a high-speed small compound molecular pump, which can be used to investigate the pumping performance of bleed blades and compression channels in a thin air environment. On the basis of the aerodynamic model, the NNIA multi-objective optimization algorithm is presented to optimize the structural parameters of the compound molecular pump. After structural parameter optimization, the maximum flow rate and compression ratio of the compound molecular pump are increased by 13.6% and 41.6%, respectively. The experimental results of the pumping performance show that the predicted data of the aerodynamic model are in good agreement with the experimental data, with an error of 12–27%. Namely, the established aerodynamic model has high accuracy and the optimized structural parameters of the compound molecular pump can provide basic conditions for the large-scale application and promotion of portable mass spectrometers.

## 1. Introduction

Vacuum pump refers to a device or equipment that uses mechanical, physical, chemical, or physicochemical methods to extract air from the container being pumped and obtain a vacuum environment. Many researchers have conducted a lot of studies on vacuum pumps: Li Zhang et al. [1] designed a conical rotor screw vacuum pump with more superior performance and adjustable flow field clearance. Jun Wang et al. [2] optimized the new rotor of the claw vacuum pump, optimized the performance of the vacuum pump, and effectively alleviated the adverse effects of the mixing process. Chunji Ren et al. [3] studied the dynamic characteristics of the multi-jaw rotor claw vacuum pump and revealed the influence of the number of claws on the performance of the vacuum pump. As a kind of vacuum pump, molecular pumps also were widely used in various fields of high-vacuum environments. Li, B. et al. [4] studied the flow rate performance of a dry pump and obtained the influence of pipe geometry on the flow rate performance of a molecular pump.

The molecular pump is an important part of the mass spectrometer [5]; the portable mass spectrometer is an important attempt for high-end analytical equipment to get out of the laboratory. As the core component of the portable mass spectrometer vacuum system, the small molecular pump accounts for the largest weight and power consumption in its system, which plays a decisive role in the miniaturization of the mass spectrometer. The existing form of compound molecular pumps is basically a combination of vertical bleed channels and compression channels. The axial direction of pumping of the vertical bleed channels is gradually pumped by a combination of multi-stage bleed blades. This combined form of bleed channel has high requirements for the axial and radial dimensions of the mass spectrometer. As the only high-speed component in a portable mass spectrometer, the small compound molecular pump can operate at a maximum speed of 90,000 rpm. The miniaturization constraint will result in an obvious decrease in the size of the molecular pump and greatly reduce its working line speed. Then, the pumping rate and compression ratio of the molecular pump are significantly reduced. Therefore, the performance analysis and structural optimization of high-speed small compound molecular pumps are crucial for the development of portable mass spectrometers.

A certain number of studies on molecular pumps have been carried out by many researchers. As for structure design, Han et al. [6] designed a five-axis magnetic levitation turbomolecular pump with a diameter of 350 mm from the perspectives of mechanical structure, thermal analysis, and electromagnetic structure. The modal characteristics of the rotor system had been improved to increase stability. The results of performance testing indicated that the designed molecular pump had a maximum flow rate of 4190 L/s and stable operating performance. Giors et al. [7] designed a spiral compression stage rotor structure to replace the disc compression stage rotor structure. This molecular pump could realize a rated speed of 42,000 rpm and a flow rate of 700 L/s. The spiral compression structure had the advantages of small leakage and compact axial size. Nasab et al. [8] designed a stepped molecular pump structure based on the Holweck-type compression molecular pump. The pumping performance of the molecular pump was tested through DSMC and improved Sickafus methods. The results showed that this structure could effectively improve the compression ratio of the molecular pump. Zhang et al. [9] established a model for the strength, mode, and electromagnetic field of the magnetic bearing and motor rotor system of a magnetic levitation molecular pump with a rotational speed of 21,000 rpm. Through multi-objective optimization algorithms, the structure size of the magnetic bearing and motor were optimized to improve the pumping performance. Most of the compound molecular pumps in the above studies were large-caliber molecular pumps with large volume and mass, whose operating speeds were mostly below 60,000 rpm. There is few research on the structural design and pump performance of compound molecular pumps under high speed and small volume constraints.

As for the simulation analysis pumping performance of a molecular pump, there are various numerical simulation methods for thin-air aerodynamics. The molecular simulation methods based on gas molecular models focus on the motion process of individual gas molecules, and the motion states of each gas molecule at different time steps are recorded. The overall behavior of gas molecules is analyzed using statistical methods, and the macroscopic gas behavior is explained from a microscopic perspective. Representative methods based on gas molecular models include molecular dynamics (MD) and Monte Carlo methods [10,11]. In addition, mesoscopic simulation models between the continuity principle and molecular model can also be used for numerical simulations of thin-air aerodynamics. The typical method includes the lattice Boltzmann method (LBM) [12]. Jou et al. [13] compared and analyzed the predictive effects of the CFD method and DSMC method on the pumping performance of the developed compound molecular pump. The results indicated that the CFD method had higher prediction accuracy when the Kn value was small, while the DSMC method had higher prediction accuracy when the gas Kn value was large. Sun et al. [14,15] proposed a predictive model for the pumping performance of compound molecular pumps based on the TPMC method. This model could obtain the flow field characteristics and pumping performance during the pumping process of compound molecular pumps based on the particle motion state. Kersevan et al. [16] introduced the application of the TPMC method and angle coefficient method in high-vacuum equipment simulation. Compared to the angle coefficient method, the TPMC method had higher solving efficiency and computational accuracy. Naris et al. [17] proposed a dynamic modeling method for the compound molecular pump, which was suitable for a conical Holweck compression stage rotor. The effects of some factors on the pumping performance of the molecular pump were analyzed, such as gas type, ambient temperature, and pre-stage pressure. Li et al. [18] compared the pumping performance of the two-dimensional model, the ideal three-dimensional model, and the actual three-dimensional model of the compound molecular pump using the DSMC method. The comparison results showed that the actual three-dimensional model had higher accuracy. Sengil et al. [19] improved the existing two-dimensional parallel DSMC solver to reduce statistical errors and shorten calculation time by dynamically changing the proportion of gas molecules. The results showed that the efficiency of the DSMC method in solving molecular pump performance had been obviously improved. Due to the high vacuum level in the working environment of the compound molecular pump, the numerical simulation method based on the continuous flow model has certain limitations and cannot directly interpret the gas motion under the molecular flow state. The Monte Carlo method (MC) based on molecular models starts from a microscopic perspective. Through the simulation of a large number of molecules, it can effectively characterize the gas motion state under transition flow and molecular flow state, which has high feasibility in the simulation of compound molecular pumps.

About the optimization of pumping performance, Svichkar et al. [20] pointed out that the structure, control methods, pre-vacuum equipment, and system gas leakage had a significant impact on the pumping performance of molecular pumps. A selection method of pre-pump was proposed to improve pumping performance. Hsieh et al. [21] pointed out that the starting pressure of the molecular pump had a significant impact on the compression ratio and pumping rate. The excessive pressure in the front stage would lead to unstable operation of the molecular pump. Sadegh et al. [22] established a simulation model for the compression stage rotor of a compound molecular pump based on the Monte Carlo method. When the shape of the spiral groove cross-section was rectangular, semi-circular, semi-elliptical, and composite-shaped, the compression ratio performance in different concentrations of gas environments was analyzed. The research results indicated that, compared to other helical cross-sections, semi-elliptical helical grooves could achieve a higher compression ratio of molecular pumps. Demikhov et al. [23] proposed an algorithm to determine the optimal number of flow channels for molecular pump bleed blades to improve pumping performance. Huang et al. [24], considering multiple physical field constraints such as mechanical strength, rotor dynamics, electromagnetic losses, and thermal characteristics, proposed a multidisciplinary optimization method for magnetic levitation molecular pumps. The above research indicates that the pumping performance of compound molecular pumps is influenced by factors such as the front-end equipment, working environment, and pumping channel structure. In the same working environment, the pumping performance of compound molecular pumps is directly determined by the structural parameters of the gas channel. However, in the process of optimizing the structural parameters of molecular pumps, the interaction between multiple structural parameters is often ignored. It is difficult to achieve the optimal combination state only by adjusting a single parameter.

To address the problems of low strength and insufficient pumping performance of molecular pumps under miniaturization constraints, this paper proposes a compound pump consisting of a horizontal bleed channel and multi-stage spiral compression channel. The pumping principle of the compound molecular pump is analyzed to obtain its preliminary structural parameters. The test particle Monte Carlo method is presented for establishing an aerodynamic model for a high-speed small compound molecules pump, which can be used to investigate the pumping performance of bleed blades and compression channels in a thin air environment. On the basis of the aerodynamic model, the NNIA multi-objective optimization algorithm is presented to optimize the structural parameters of the composite molecular pump. A pumping performance experiment is carried out to evaluate the prediction accuracy of the aerodynamic model and the effectiveness of the optimized structure parameters.

## 2. The Pumping Principle and Preliminary Structure Design

### 2.1. Pumping Principle

The portable mass spectrometer has the characteristics of light weight, portability, and real-time detection, and it has strict limitations on the size and weight of components. As the core component of the portable mass spectrometer vacuum system, the small molecular pump accounts for the largest weight and power consumption in its system, which plays a decisive role in the miniaturization of the mass spectrometer. The performance requirements of the high-speed small compound molecular pump in this study are shown in Table 1.

**Figure 1:**
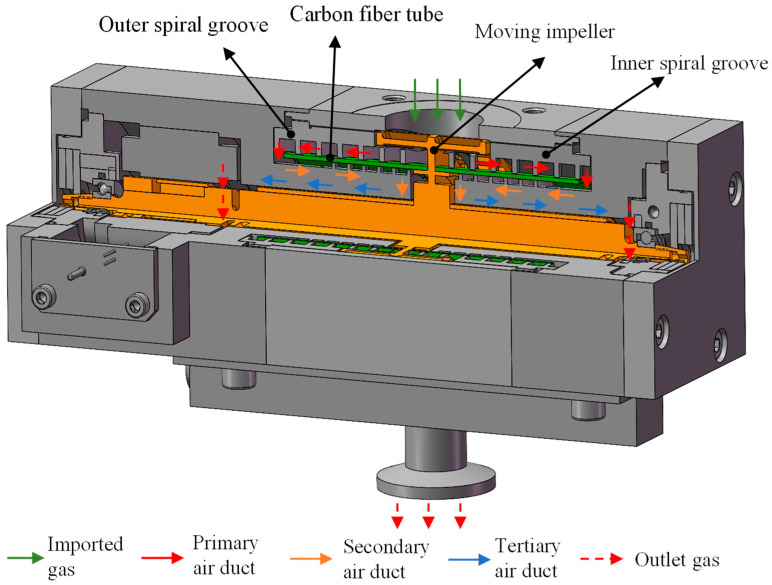
The structural diagram of the high-speed small compound molecular pump pumping unit.

The high-speed small compound molecular pump is mainly composed of a pumping unit, support unit, and driving unit. The main function of the pumping unit is to pump gas from the high-vacuum environment to the previous low-vacuum environment. The main function of the support unit is to ensure the stable operation of all components at high speeds. The main function of the driving unit is to provide a stable power source for the molecular pump and ensure the stability of the rotor speed of the system during normal operation. The structural diagram of the high-speed small compound molecular pump pumping unit is shown in Figure 1. To meet the requirements of high-speed small compound molecular pumps with good pumping performance under limited weight and size, a combination of bleed stage rotor and compression stage rotor is adopted in this pumping unit. The symmetrical layout is applied to fully utilize the internal space of the molecular pump. A horizontal bleed channel form is adopted in this bleed stage rotor. Compared to the vertical bleed rotor, the advantage of the horizontal bleed rotor is smaller volume. The direction of gas movement during operation is perpendicular to the rotor’s rotational axis, which makes the blade load more uniform, the stress state better, and the operating life longer. The bleed rotor is directly connected to the vacuum chamber. Gas molecules pass through the inlet and enter the compression stage rotor after obtaining a high flow rate through the bleed stage rotor. The compression stage rotor is composed of three spiral channels, which form a small gap channel between the carbon fiber cylinder and the inner and outer groove cylinders. The size of the spiral channel gradually decreases and the degree of compression of the gas gradually increases. Then, the gas flows in a directional direction along the channel. Finally, the gas is discharged into the low-vacuum environment of the previous stage through both sides of the bearing. The multi-stage compression channel enables the molecular pump to maintain good pumping performance under the high front stage pressure and provides a stable high compression ratio for the molecular pump. The design of compound molecular pumps in this study mainly includes the overall rotor structure and interstage arrangement. The design parameters of the bleed stage rotor include the inner and outer diameters and heights of the impeller, the inclination angle, and the number of blades. The design parameters of the compression stage rotor include the inner and outer diameter and height of the spiral groove barrel, compression clearance, spiral rise angle, and groove depth.

### 2.2. Preliminary Structure Design

(1) Bleed stage rotor

**Figure 2:**
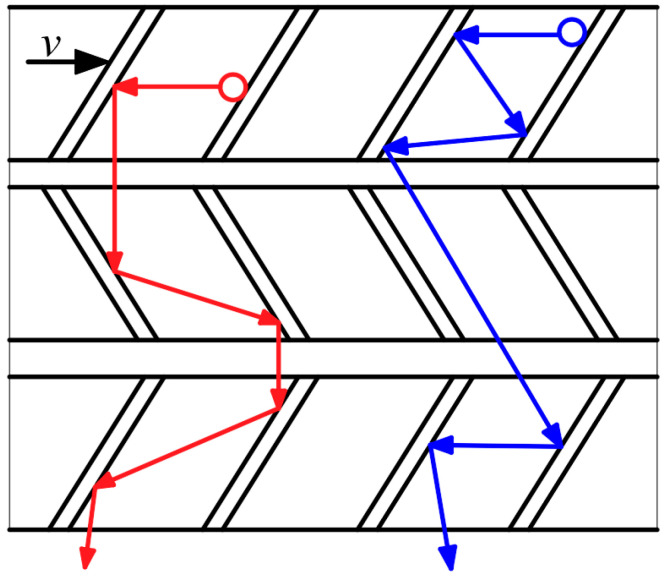
The pumping principle of bleed stage rotor.

The bleed stage rotor relies on the collision between the high-speed rotating bleed blades and the gas molecules in the vacuum chamber to transfer momentum. The pumping effect is formed by forcing gas molecules to move forward towards the outlet. In order to fully utilize the internal space of the molecular pump and improve its pumping capacity, the bleed stage rotor is set up with a symmetrical pumping structure. In order to enhance the strength of thin bleed blades at high speeds, an integrated impeller shaft is formed by connecting the bleed blades to the main shaft. The multi-stage blade form of the bleed stage rotor is shown in Figure 2. It can be seen that this multi-stage blade is composed of multiple single-stage blades connected in series. The pumping principle of multi-stage blades is similar to that of single-stage blades, whose pumping performance can be regarded as the superposition of multiple single-stage bleed blades.

**Figure 3:**
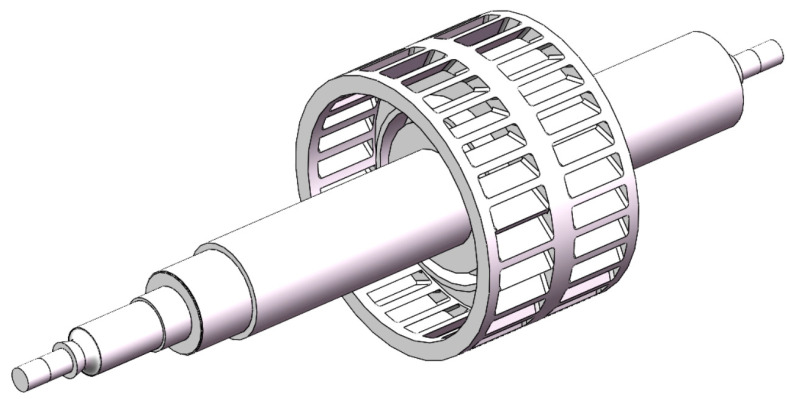
The schematic diagram of the bleed stage rotor pumping structure.

According to the pumping principle and installation conditions, after theoretical calculation, the schematic diagram of the bleed stage rotor pumping structure is shown in Figure 3. The structural parameters and theoretical pumping performance of the bleed stage rotor are shown in Table 2. The theoretical flow rate (S) of the bleed stage rotor is 8.44 L/s, which is 6 L/s higher than the flow rate (6 L/s) of design requirement.

(2) Compression stage rotor

**Figure 4:**
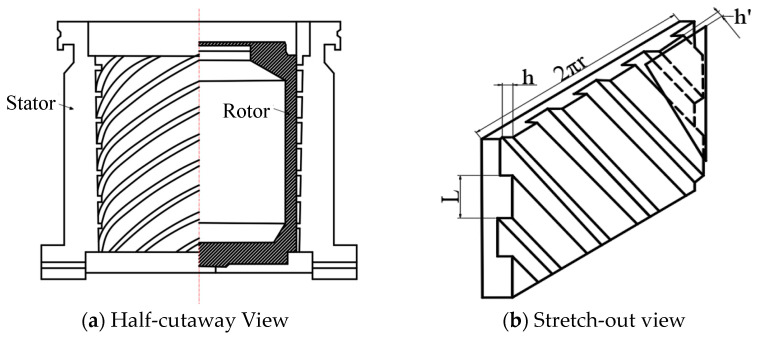
The structural diagram of the compression stage rotor.

The compression stage rotor is a Holweck-type compression structure, consisting of a shaft (rotor) and an outer cylinder (stator). There are spiral grooves on the rotating shaft and the gas in the pump is directionally transmitted through the spiral channel. The structural diagram of the compression stage rotor is shown in Figure 4, where h′ is the gap between the rotor and stator. L is the width of the spiral channel.

**Figure 5:**
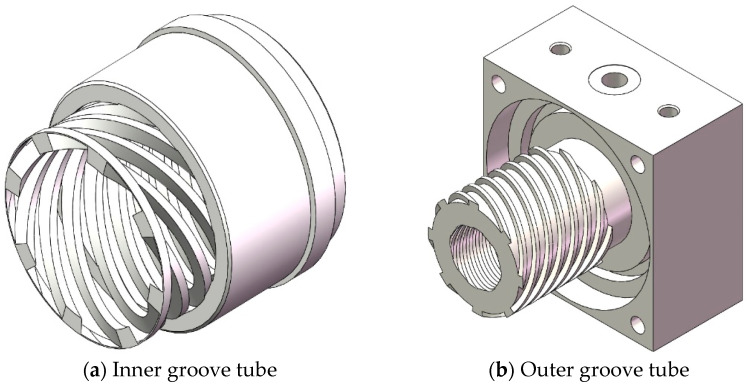
The structure diagram of the compression stage stator.

In order to meet the requirements of small size and light weight, the compression stage rotor adopts a multi-stage spiral channel structure. The multi-stage spiral channel is achieved through ultra precision assembly between the molecular pump rotor and stator structure. The rotor structure includes the main shaft and carbon fiber tube. The stator structure consists of the inner and outer groove tubes. The structure diagram of the compression stage stator is shown in Figure 5.

According to the pumping principle and installation conditions, after theoretical calculation, the structural parameters and theoretical pumping performance of the multi-stage helical channel of the compression stage rotor are shown in Figure 3. The multi-stage spiral channels can be regarded as a series connection of multiple single-spiral channels. Therefore, its compression ratio is equal to the product of the compression ratios of each part. The theoretical compression ratio of the designed compression stage rotor is *C* = 1.42 × 10^7^, which is far higher than the required compression ratio (1 × 10^6^). The structural parameters and theoretical pumping performance of the multi-stage helical channel are listed in Table 3.

## 3. The Simulation of Pumping Performance

### 3.1. Aerodynamic Model in Thin Air Environment

The working environment of the high-speed small compound molecular pumps is generally in transition state and molecular watersheds (Kn > 1) due to high vacuum and thin gas. The continuity simulation method based on the continuity assumption makes it difficult to explain the gas motion state in this state. The Monte Carlo method based on molecular models can simulate the gas motion state under high vacuum. This method characterizes a large number of microscopic gas molecules using gas molecule samples and obtains the macroscopic pumping performance through statistical methods. At present, Monte Carlo methods mainly include the direct simulation Monte Carlo method (DSMC) and test particle Monte Carlo method (TPMC). The DSMC method introduces the gas collision model to determine the molecular motion state after collision with a certain probability. The TPMC method only considers the collision between gas molecules and the wall of the vacuum chamber. It is more suitable for the characteristics of gas molecules in the state of molecular flow due to the relatively low probability of intermolecular collision. To some extent, the TPMC method can be treated as an improvement of the DSMC method in molecular flow states, which can make the Monte Carlo method more suitable for the gas motion characteristics under molecular flow states. COMSOL multi-physics coupling simulation software is used for calculation and solution. In COMSOL 6.0 software, the particle tracking module is used to implement the TPMC method. The specific implementation method is as follows: (1) a representative gas molecule sample is established. (2) After the sample is released, the motion characteristics of each gas molecule in each time step are recorded to obtain the motion process of all gas molecules. (3) The specific statistical parameters are used to characterize macroscopic physical quantities. When using the TPMC method for simulation analysis, gas molecules follow Newton’s second law during their motion, as shown in Equation (1):

(1) $$\frac{d}{dt}\left(m_{p}\frac{dv}{dt}\right)=F_{t}$$

where *m*_p_ is the molecular weight of the gas. ***v*** is the velocity vector. ***F***_t_ is the total force exerted on gas molecules.

To describe the high-speed rotation motion characteristics of small compound molecular pump, the simulation domain of gas molecules should be set as the rotation domain. Then, relative to the fixed reference frame, the reference frame of gas molecules is in a high-speed rotating state. The aerodynamic model is established in a non-inertial reference frame. Therefore, some hypothetical forces should be applied to gas molecules in simulation calculations, including centrifugal force, Coriolis force, and Euler force, as shown in Equations (2)–(5):

(2) $$F_{\mathrm{ce}}=m_{p}\omega \times \left(\omega \times r\right)$$

(3) $$F_{\mathrm{cr}}=2m_{p}\omega \times v$$

(4) $$F_{o}=m_{p}\dot{\omega }\times r$$

(5) $$\frac{d\left(m_{p}v\right)}{dt}=F_{\mathrm{ce}}+F_{\mathrm{cr}}+F_{o}$$

where ***F***_ce_ is centrifugal force. ***F***_cr_ is Coriolis force. ***F***_o_ is Euler force. ***ω*** is the rotational angular velocity of a rotating reference frame.

**Figure 6:**
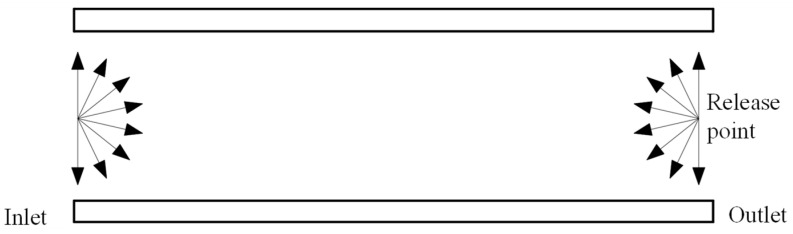
The boundary condition of inlet and outlet.

In the simulation model of the pumping channel in a small compound molecular pump, considering rotational symmetry, the gas molecules are released in the designated inlet and outlet planes, as shown in Figure 6. Moreover, the release points of gas molecules are evenly distributed on the inlet and outlet surfaces. The motion velocity of gas molecules after release follows the Maxwell rate distribution, as shown in Equation (6).

(6) $$F\left(v\right)=\sqrt{\frac{2}{\pi }}{\left(\frac{m}{kT}\right)}^{\frac{3}{2}}v^{2}e^{-\frac{mv^{2}}{2kT}}$$

In the simulation model, the adsorption and desorption of gas molecules on the wall follow Knudsen’s cosine law, and the temperature condition is the gas temperature. That is, after the collision between gas molecules and the wall, the direction of gas molecules’ movement when leaving the wall is independent of the direction of the incident angle, as shown in Equation (7):

(7) $$dN_{\theta }=\frac{n\bar{v}}{4\pi }\cos\theta \cdot d\omega \cdot ds$$

where d*N*_θ_ is the total number of gas molecules impacting a unit area per unit time. d*ω* is the solid angle. d*s* is the area unit. *θ* is the normal angle between the solid angle and area unit.

### 3.2. The Simulation and Analysis of Bleed Stage Rotor

**Figure 7:**
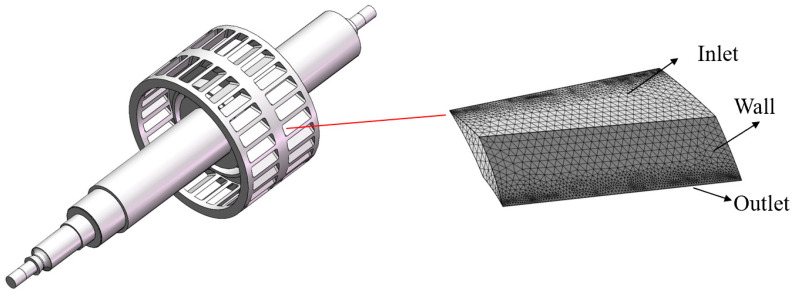
The simulation model and boundary condition of the bleed stage rotor.

The simulation model and boundary conditions of the bleed stage rotor are shown in Figure 7. Multiple narrow blades are evenly distributed on the circumference at the same inclination angle. The pumping channels of each blade are consistent and there is periodicity and symmetry in the structure of all blades. Therefore, in order to improve computational efficiency, a single gas channel formed by a pair of adjacent blades can be selected as the object of simulation research. The blade parameters are as follows: the blade inclination angle is 30°, the pitch to chord ratio is 1, and the blade spacing is 4 mm.

**Figure 8:**
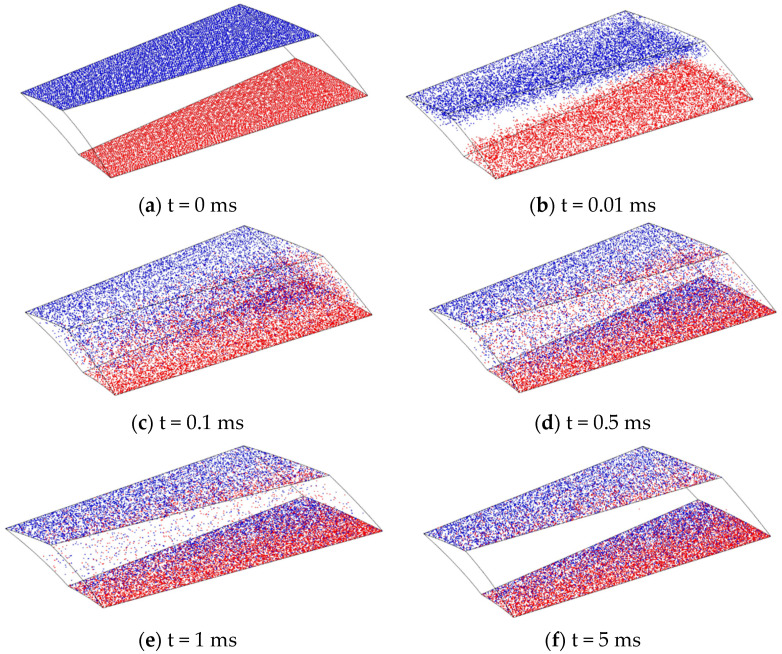
The simulation results of single blade channel at the speed of 90,000 rpm.

According to the above simulation settings, the finite element simulation results of a single blade channel of a bleed stage rotor at the speed of 90,000 rpm are shown in Figure 8. This transient simulation model can intuitively display the “transport” effect of gas molecules during bleed blade operation, elucidating the working principle of the bleed stage rotor. From Figure 8, it can be seen that, with the heat release of gas molecules, the gas molecules released from the outlet and inlet both have a trend of moving towards the opposite side, as shown in Figure 8b. However, as the movement process progresses, gas molecules collide with the blade wall, which lead to gas molecules gradually forming a trend of aggregation towards the outlet surface. After multiple collisions with the wall, most gas molecules aggregate on the outlet surface (as shown in Figure 8f). Namely, most of the gas molecules released from the inlet can reach the outlet surface, while a small portion of the gas molecules released from the outlet can reach the inlet surface. From a macro perspective, gas molecules form a forward transportation, achieving a pumping effect. In Figure 8, the blue particles are the particles released at the entrance, and the red particles are the particles released at the exit.

The Box–Behnken design (BBD) method is used to design the structural parameters of bleed stage rotor, including blade inclination angle (*α*), blade spacing (*a*), and blade chord length (*b*). The flow rate and compression ratio are selected as the response targets. The coding and level of the structural parameters of the bleed stage rotor are shown in Table 4. According to the aerodynamic model and BBD method, the design scheme of bleed stage rotor structural parameters and its pumping performance can be obtained, as shown in Table 5.

### 3.3. The Simulation and Analysis of Compression Stage Rotor

**Figure 9:**
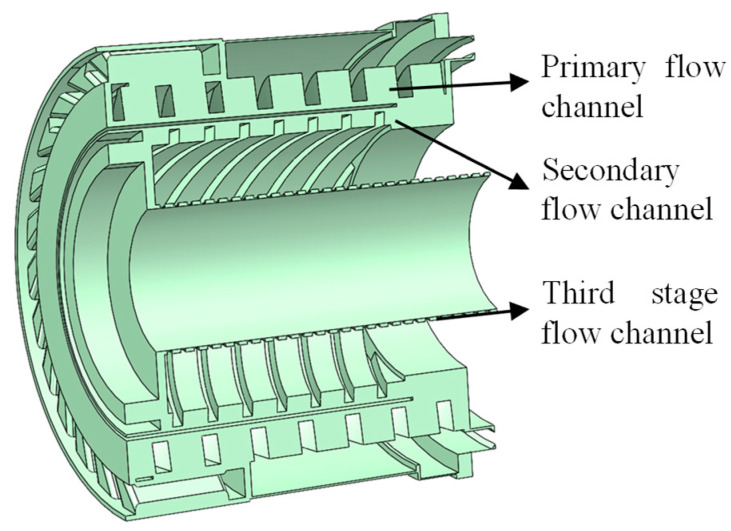
The multi-stage spiral gas compression channel of the compression stage rotor.

The multi-stage spiral gas compression channel used in the compression stage rotor of the small compound molecular pump is shown in Figure 9. The structural parameters are as follows: the diameter of the spiral groove is 32 mm, the depth of the spiral groove is 2 mm, the helix angle is 30 °, the gap between adjacent spiral grooves is 0.15 mm, the length of the compression cylinder is 43 mm, and the number of spiral groove heads is 8. The multi-stage gas channels are tightly connected to each other. However, due to the significant differences between the three stages of channels, the simulation analysis of the overall compression stage rotor is difficult. For the convenience of simulation analysis, the multi-stage channels are divided into multiple single-stage channels, and each stage channel is simulated separately. The overall pumping performance of the compression stage rotor can be obtained by superposing multiple single-stage channels.

**Figure 10:**
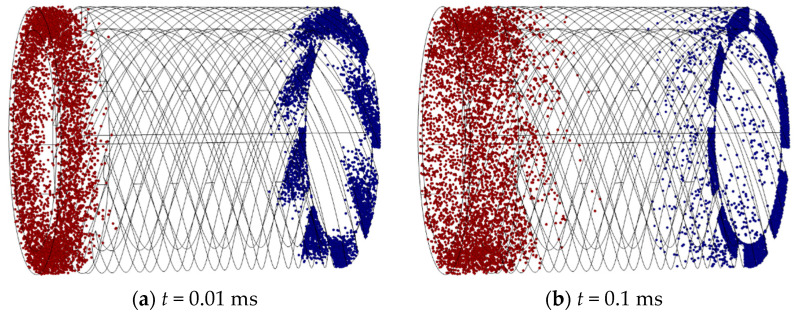

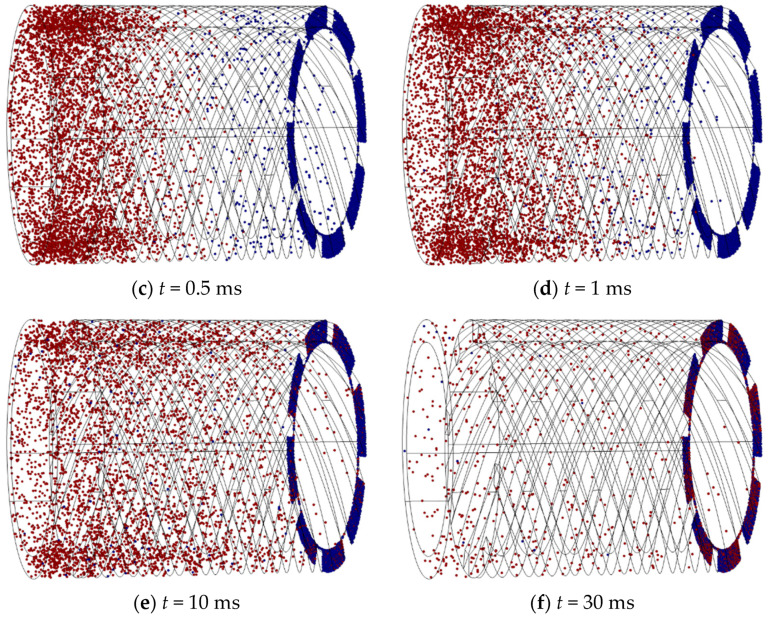
The simulation results of single-stage compression channel at the speed of 90,000 rpm.

According to the above simulation settings, the finite element simulation results of the single-stage compression channel of the compression stage rotor at the working speed of 90,000 rpm can be obtained, as shown in Figure 10. This simulation model can visually depict the motion trajectory of gas molecules in the rotor channel of the compression stage and can explain why the compression stage rotor can improve the resistance of small compound molecular pumps to pressure disturbances in the previous stage. The vast majority of gas molecules released from the inlet surface will collide with the spiral channel and reach the outlet surface. However, a small amount of gas molecules released from the outlet surface reach the inlet surface through interstitial motion. This phenomenon is known as “clearance regurgitation”, which reduces the suction effect of the compression channel. From Figure 10, it can be seen that the gas molecules released from both the outlet and inlet surfaces tend to move towards the opposite direction (as shown in Figure 10a). Although most of the gas molecules move towards the outlet surface under the guidance of the spiral grooves, there are still some gas molecules that move towards the inlet surface, resulting in some gap leakage issues. Most gas molecules go through multiple collisions and reflections with the rotor surface and channel wall while passing through the spiral channel. Due to the guiding effect of the spiral channel, after multiple reflections and releases of gas molecules, the original motion trend of gas molecules released from the inlet surface will be strengthened and the original motion trend of gas molecules released from the outlet surface will be suppressed. It means that most gas molecules will move in a direction towards the outlet surface in the spiral channel (as shown in Figure 10f). From a macro perspective, this phenomenon indicates that molecular pumps equipped with compression stage rotors can operate normally at higher front-stage pressures. In Figure 10, the red particles are the particles released at the entrance, and the blue particles are the particles released at the exit.

The Box–Behnken design (BBD) method is used to design the structural parameters of the compression stage rotor, including gap distance (h′), spiral groove depth (h), and spiral rising angle (*φ*). The flow rate and compression ratio are selected as the response targets. The coding and level of the structural parameters of the compression stage rotor are shown in Table 6. According to the aerodynamic model and BBD method, the design scheme of the structural parameters of the compression stage rotor and its pumping performance can be obtained, as shown in Table 7.

## 4. The Optimization of Structure Parameters

### 4.1. Interaction between Structural Parameters

(1) Bleed stage rotor

In order to observe the degree of influence of different test factor levels on the test results and the primary and secondary order more intuitively and analyze the influence of changes in the structural parameters of small complex molecular pumps on the pumping performance, the curved surface response analysis method was used to perform quadratic multiple regression analysis and fitting of the compression ratio and pumping rate under different structural parameters of small molecular pumps. The relationship between the level of different structure parameters and the pumping performance is obtained, respectively.

**Figure 11:**
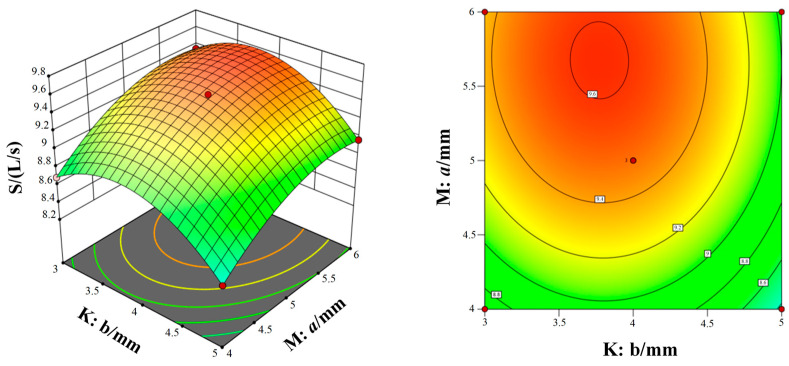
The interaction of blade spacing and blade chord length on the flow rate.

**Figure 12:**
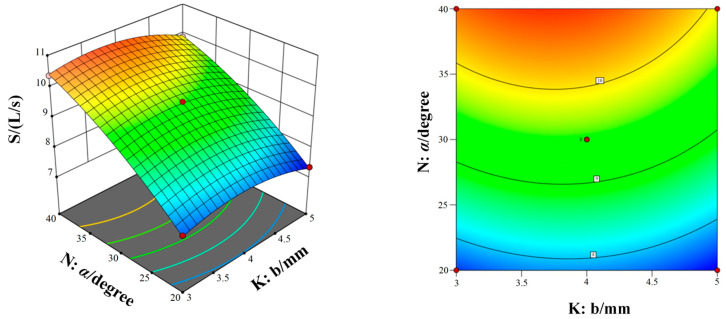
The interaction of blade inclination angle and blade chord length on the flow rate.

**Figure 13:**
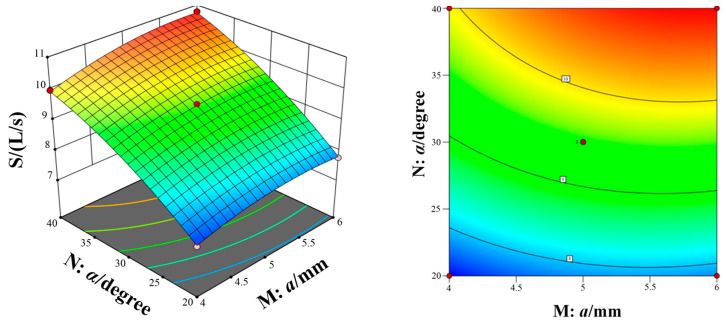
The interaction of blade spacing and blade inclination angle on the flow rate.

Figure 11, Figure 12 and Figure 13 show the response surfaces of the interaction between the other two structural parameters to the bleed flow rate when any factor is at the center value. According to Figure 11, Figure 12 and Figure 13, it can be concluded that blade spacing, blade chord length, and blade inclination angle have different influences on the flow rate of compression stage. With the increase in blade spacing, the flow rate first increases and then decreases and, with the increase in blade chord length and blade inclination angle, the flow rate also increases gradually. In addition, Figure 11 shows the interaction effect of blade spacing (K) and blade chord length (N) on the flow rate. It can be seen that there is a highest point on the response surface, which means there is a maximum flow rate within this range. The contour map in Figure 11 is elliptical, which indicates there is a significant interaction between blade spacing and blade chord length. Meanwhile, the interaction between parameters in Figure 12 and Figure 13 is not significant. Taking into account the three response surfaces of the flow rate of the bleed stage rotor, it can be seen that the flow rate changes sharply along the blade inclination angle. It means that the blade inclination angle is the main influence factor of the flow rate. The structural parameters for obtaining the maximum flow rate of the bleed stage rotor based on the response surface are b = 3.71 mm, a = 5.88 mm, and α = 39.98°, with a compression ratio of C = 2.15 and a flow rate of S = 10.78 L/s.

**Figure 14:**
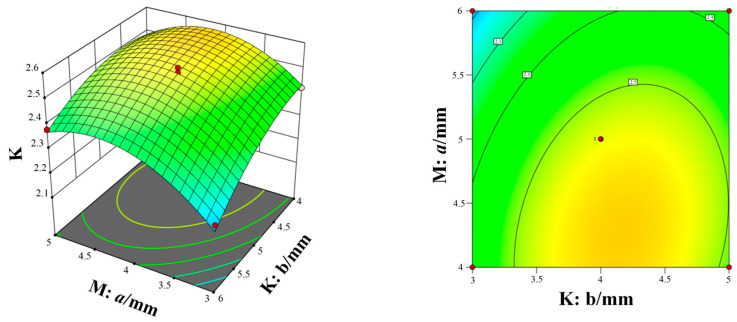
The interaction of blade spacing and blade chord length on the compression ratio.

**Figure 15:**
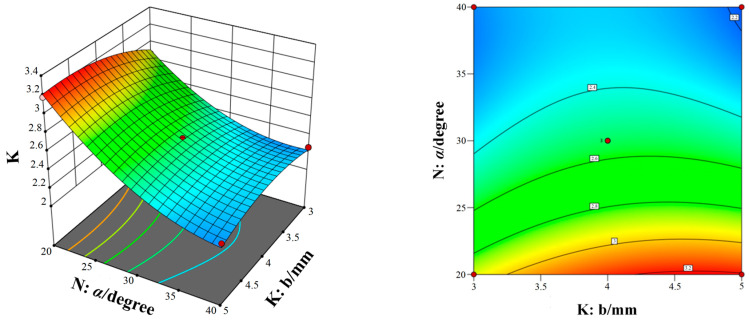
The interaction of blade inclination angle and blade chord length on the compression ratio.

**Figure 16:**
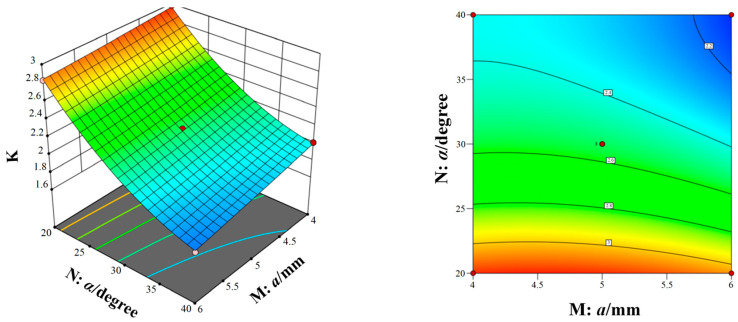
The interaction of blade spacing and blade inclination angle on the compression ratio.

Figure 14, Figure 15 and Figure 16 show the response surfaces of the interaction between the other two structural parameters to the bleed compression ratio when any factor is at the center value. According to Figure 14, Figure 15 and Figure 16, it can be concluded that blade spacing, blade chord length, and blade inclination angle have different influences on the compression ratio of compression stage. With the increase in blade chord length, the compression ratio first increases and then decreases, while the compression ratio gradually decreases as blade inclination angle and blade spacing increases. In addition, Figure 14 shows the interaction effect of blade spacing (K) and blade chord length (M) on the compression ratio. It can be seen that there is a highest point on the response surface, which means there is a maximum compression ratio within this range. The contour map in Figure 14 is elliptical, which indicates there is a significant interaction between blade spacing and blade chord length. Meanwhile, the interaction between parameters in Figure 15 and Figure 16 is not significant. Taking into account the three response surfaces of the compression ratio of the bleed stage rotor, it can be seen that the compression ratio changes sharply along the blade inclination angle and blade chord length. It means that the blade inclination angle and blade chord length are the main influence factors of the compression ratio. The structural parameters for obtaining the maximum compression ratio of the bleed stage rotor based on the response surface are b = 4.60 mm, a = 4.67 mm, and α = 20.01°, with a compression ratio of C = 3.23 and a flow rate of S = 7.5 L/s.

(2) Compression stage rotor

Using an interaction analysis method and surface response method, the structural parameters for the maximum single response target can be obtained, as shown in Table 8. From Table 8, it can be seen that there is a significant difference in the optimal parameter values that only meet the maximum compression ratio or maximum flow rate. It is not possible to obtain the optimal design value for comprehensive performance through direct optimization. It is necessary to adopt multi-objective optimization methods to obtain the optimal parameter combination that takes into account compression ratio and compression rate.

### 4.2. Multi-Objective Optimization of Structural Parameters

To improve the pumping performance (high compression ratio and flow rate) of a high-speed small compound molecular pump, based on the regression model from response surface methodology, Non-normalized Neighbor Immune Algorithm (NNIA) is presented for multi-objective optimization to obtain the optimal structural parameters of the bleed stage rotor and compression stage rotor, as shown in Equation (8) [25]:

(8) $$F\left(x\right)=\max\left\{C\left(b,a,\alpha ,h^{\prime },h,\varphi \right),S\left(b,a,\alpha ,h^{\prime },h,\varphi \right)\right\}$$

**Figure 17:**
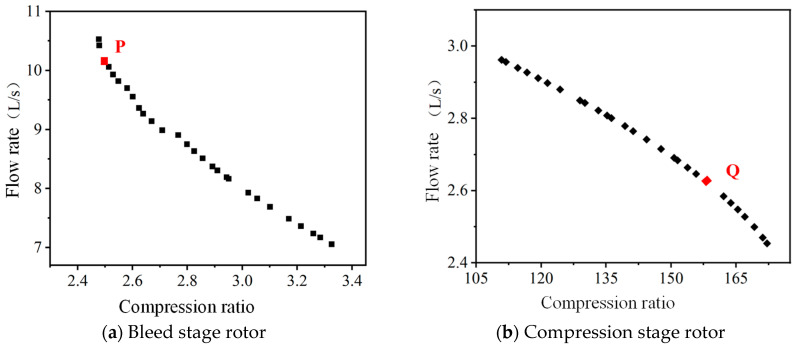
The Pareto solution set for the structural parameters of compound molecular pump.

Based on NNIA multi-objective optimization algorithm, the Pareto solution set of structural parameters of the bleed stage rotor and compression stage rotor can be obtained, as shown in Figure 17. The Pareto solution set is a set of effective solutions under given conditions, where the solutions within the set are nondominant and can be treated as the equilibrium point for multi-objective optimization. Compared to other solutions, these nondominant solutions have the least related goal conflicts and can provide a larger selection interval for the optimization object. From Figure 17, it can be seen that there is a negative correlation between the compression ratio and flow rate of the bleed stage rotor and the compression stage rotor. It means that there is a certain conflict in the design values for optimal performance.

In practical work, a compound molecular pump requires both high flow rate and high compression ratio, and the corresponding comprehensive optimal solution is selected from the Pareto solution set. For small compound molecular pumps, the design purpose of the bleed stage rotor is to provide a larger compression speed. The design purpose of the compression stage rotor is to provide a large compression ratio. Therefore, the P point with a higher compression rate is selected as the final design scheme for the bleed stage rotor structure. The Q point with a larger compression ratio is chosen as the final design scheme for the compression stage rotor structure, as shown in Table 9.

To facilitate the manufacturing and assembly of compound molecular pumps, it is necessary to round off the structural parameters of the optimized molecular pump. Meanwhile, in order to verify the effectiveness of the optimized and rounded structural parameters, based on the aerodynamic simulation model, the pumping performance of the compound molecular pump before and after optimization can be obtained, as shown in Table 10. From Table 10, it can be seen that the pumping performance of the optimized bleed stage rotor and compression stage rotor has been improved. Through comparison, for the bleed stage rotor, the optimized structural parameters increased the flow rate by 15.6% and the compression ratio by 2.4%. For the compression stage rotor, the compression ratio is increased by 52.7% and the flow rate is increased by 6.2%. This means that the Pareto optimal solution obtained from the multi-objective optimization algorithm NNIA can effectively improve the pumping performance of small compound molecular pumps.

## 5. The Pumping Performance Experiment

### 5.1. Experiment Detail

**Figure 18:**
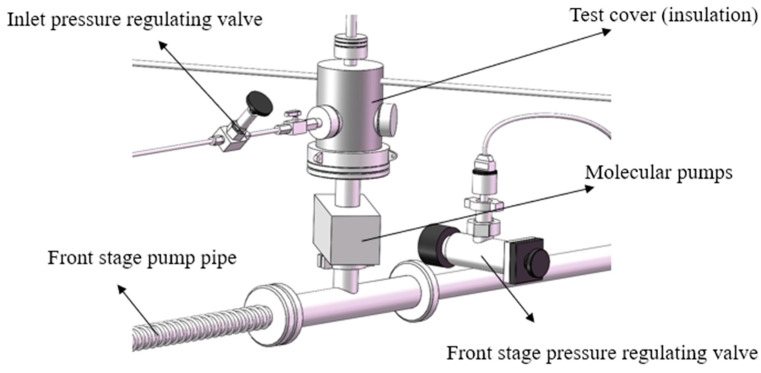
The experimental platform for testing the pumping performance of the high-speed small compound molecular pump.

The experimental platform for testing the pumping performance of the high-speed small compound molecular pump is shown Figure 18, which consists of an inlet pressure-regulating valve, front stage pump, test cover (insulation), molecular pump, front stage pressure-regulating valve, etc. The limit pressure of the front stage pump is 5 Pa. The experimental environment is 20 ℃. The relative humidity is 55~65%. The measured gas is nitrogen with a mass purity of 99.99%.

The measurement steps are as follows:

(1) Check the testing equipment and environment. Check whether the status of regulating valves, front stage pumps, molecular pumps, and other equipment is normal. Ensure that the experimental environment temperature is 20 ℃ and the relative humidity is 55~65%.

(2) Start the front stage pump; work for 0.5 h in advance. Maintain the vacuum environment of the front stage.

(3) Start the small compound molecular pump. After normal operating for 1 h, start the glass fiber heating strip, heat the test cover to 220 ℃, and bake it for 48 h.

(4) Stop baking and continue running for 48 h. During operation, the ionization vacuum gauge needs to be degassed according to the manufacturer’s regulations, thus degassing every 10 h. The final pressure inside the test cover is the limit pressure of the small compound molecular pump. The pressure at the exhaust port of the molecular pump is recorded as the previous stage pressure.

(5) Conduct the compression ratio test of small compound molecular pumps. The test gas is nitrogen with a mass purity of 99.99%. The nitrogen is filled into the exhaust port of the small compound molecular pump. Gradually increase the pressure in the front stage of the molecular pump.

(6) Observe the inlet pressure of the molecular pump and the front stage pressure. To obtain the effective compression ratio, it is necessary to ensure that the variation of the two pressures does not exceed ±5% within five minutes.

(7) Conduct the flow rate test of the small compound molecular pump. Connect nitrogen to the inlet of the gas flow meter. Slowly open the inlet pressure-regulating valve to make the gas fill into the test cover. Increase the inlet pressure.

(8) Continue to adjust the valve for gradually increasing the inlet pressure. Test three sets of data at each order of magnitude. Record the inlet pressure and the gas mass meter of each group.

(9) Replace the test gas with helium. Repeat the above steps.

### 5.2. Experimental Result

(1) Compression ratio

**Figure 19:**
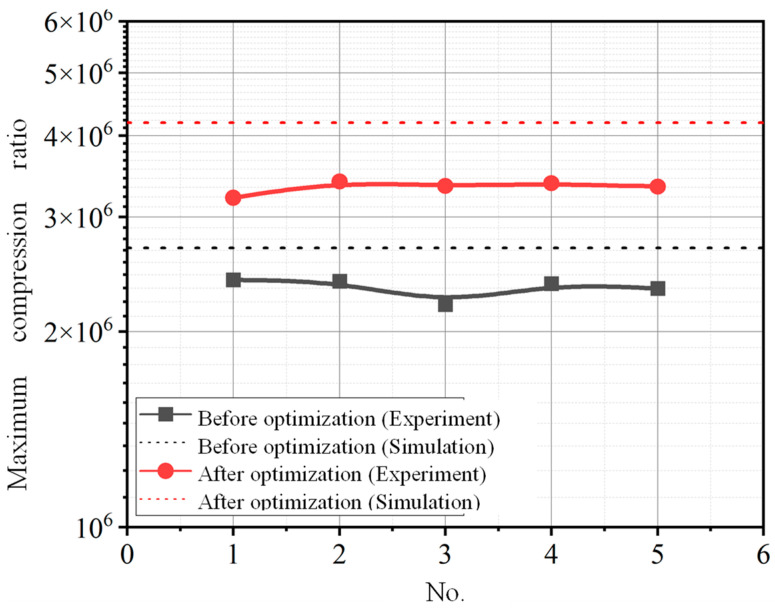
The comparison results of the experimental value and simulation predicted value of the maximum compression ratio.

The maximum compression ratio of the compound molecular pump is obtained through multiple repeated experimental measurements. The comparison results of the experimental value and simulation predicted value of the maximum compression ratio are shown in Figure 19. It can be seen that all simulation values are greater than experimental values. The relative error range between the simulation prediction results and the experimental results is 12.08~23.72%. This means that the established aerodynamic simulation model has high prediction accuracy for the compression ratio. In the actual working process of the molecular pump, the pumping unit is connected to other units through several transition devices. The impact of these transition devices on the pumping performance of molecular pumps is complex and difficult to quantify. Therefore, in the simulation model, the influence of these transition devices is ignored. In addition, the measurement instrument errors, gas leaks, and the simplified assumptions in simulation models can all lead to some errors. Therefore, there is a certain degree of error between the predicted results of the simulation model and the experimental results. However, the variation laws obtained from the simulation model are basically consistent with the experimental results. The simulation analysis models can be used to optimize the structural parameters of molecular pumps in order to achieve better pumping performance. In addition, after structural parameter optimization, the maximum compression ratio of the compound molecular pump is increased by 41.6% (from 2.4 × 10^6^ to 3.4 × 10^6^).

(2) Flow rate

**Figure 20:**
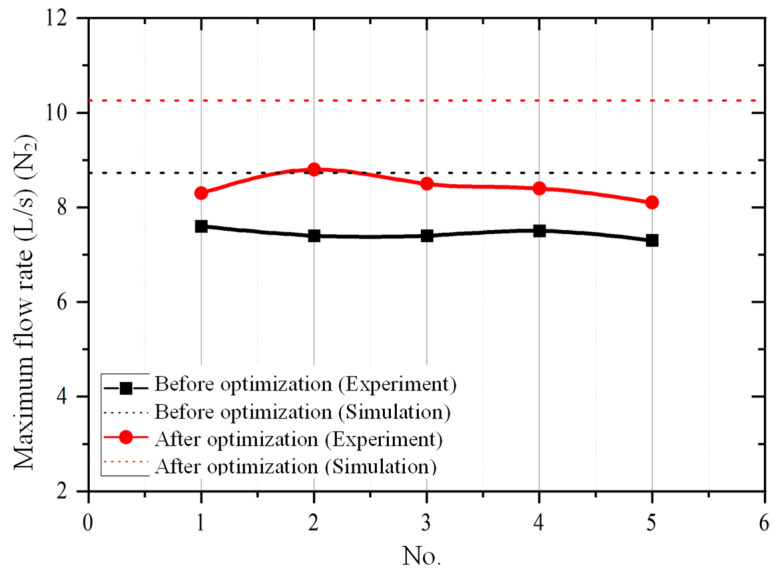
The comparison results of the experimental value and simulation predicted value of the maximum flow rate.

The maximum flow rate of the compound molecular pump is obtained through multiple repeated experimental measurements. The comparison results of the experimental value and simulation predicted value of the maximum flow rate are shown in Figure 20. It can be seen that all simulation values are greater than experimental values. The relative error range between the simulation prediction results and the experimental results is 14.86~26.67%. This means that the established aerodynamic simulation model has high prediction accuracy for the flow rate. In addition, after structural parameter optimization, the maximum flow rate of the compound molecular pump is increased by 13.6% (from 7.6 L/s to 8.8 L/s).

For the compression ratio, the relative error before optimization is 10.8–17.6% and the relative error after optimization is 15.4–19.5%. For flow rate, the relative error before optimization is 16.8–20% and the relative error after optimization is 15.3–24.4%. The relative error after optimization is slightly higher than the error before optimization. For the bleed stage rotor, the blade chord length and blade inclination angle had been increased after the optimization. For the compression stage rotor, the bleed stage and the spiral groove depth had been increased after the optimization. The increasing of these structural parameters would increase the manufacturing difficulty of the molecular pump, so as to reduce the geometric accuracy of the rotor. However, the geometric error of the rotor is ignored in the simulation model. Therefore, the difference between the experimental data and numerical data is slightly higher after the optimization than before the optimization.

(3) Limit pressure

**Figure 21:**
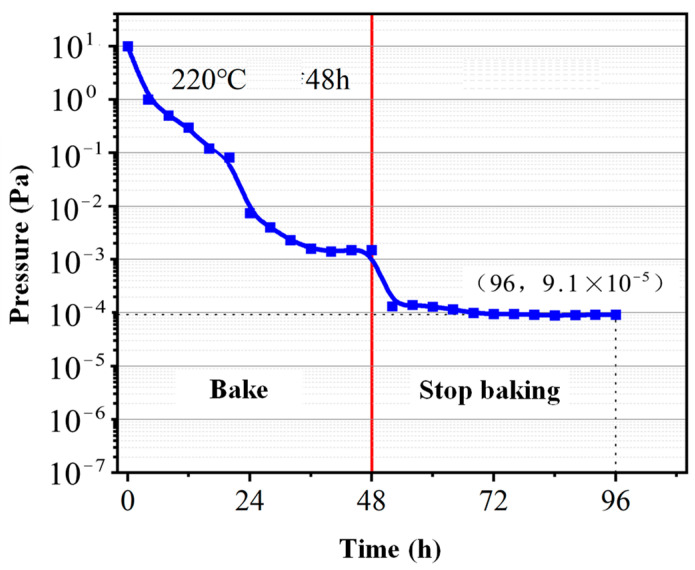
Ultimate pressure test results of small compound molecular pump.

The variation curve of the ultimate pressure of the small compound molecular pump along with the high-temperature baking and pumping time is shown in Figure 21. It can be seen that the gas pressure inside the test cover rapidly decreases under the continuous pumping and baking. After baking and pumping for 24 h, the pressure drop rate gradually decreases and finally stabilizes at 1.2 × 10^−3^ Pa. After stopping baking, the limit pressure further decreases as the temperature of the test cover decreases. This phenomenon is mainly due to the fact that the gas activity decreases with the decrease in temperature. In a limited space, the gas pressure will also decrease as the temperature decreases. After pumping for 24 h again, the gas pressure in the compound molecular pump stabilizes at 9.1 × 10^−5^ Pa.

## 6. Conclusions

This paper presents a compound pump, consisting of a horizontal bleed and multi-stage spiral compression channel, to meet the requirements of obtaining a vacuum environment under small constraints. Thin aerodynamic modeling, correlation factor analysis, multi-objective optimization, and experimental verification methods have been used to optimize the structural parameters of small molecular pumps to achieve better pumping performance.

(1) Based on the combination of the horizontal bleed channel and multi-stage spiral compression channel, the designed high-speed (90,000 rpm) and small (1.8 kg, 175 mm × 75 mm × 135 mm) compound molecular pump can achieve the maximum flow rate of 8.8 L/s, the maximum compression ratio of 3.4 × 10^6^, and the ultimate pressure of 9.1 × 10^−5^ Pa. This pumping performance is excellent among the molecular pumps of similar size and weight.

(2) The comparative analysis of the experimental and simulation results shows that the established rarefied aerodynamic model has high accuracy. The influence of molecular pump structural parameters on pumping performance can be obtained through simulation analysis. It means that this model can be used for guiding the design of a compound molecular pump.

(3) The optimized structural parameters from NNIA multi-objective optimization algorithm can increase the maximum flow rate and compression ratio of a compound molecular pump by 13.6% and 41.6%, respectively. It means that the presented optimization algorithm can provide reference for the structural optimization of a compound molecular pump.

## Figures and Tables

**Table 1:** The performance requirements of the high-speed small compound molecular pump.

Performance Index	Value
Flow rate	N_2_: >6 L/s
Compression ratio	N_2_: >1 × 10^6^
Ultimate pressure	<5 × 10^−4^ Pa
Rated rotary speed	90,000 rpm
Weight	<1.85 kg
Size	175 mm × 75 mm × 135 mm

**Table 2:** The structural parameters and theoretical pumping performance of the bleed stage rotor.

No.	Content	Value
1	Blade inclination angle	30°
2	Pitch to chord ratio	1.0
3	Teeth number	24
4	Forward transmission rate	0.5381
5	Reverse transmission rate	0.2414
6	He’s coefficient	0.297
7	Compression ratio	2.22
8	Flow rate	8.44

**Table 3:** The structural parameters and theoretical pumping performance of the multi-stage helical channel.

Content	First Stage	Second Stage	Third Stage
Compression drum length, *l*/mm	43	30	42
Compression ratio, *C* (Single stage)	169.48	141.38	297.01
Flow rate, *S*/(L/s)	2.271	1.807	0.604
Number of spiral grooves, *γ*	8	8	8
Spiral groove depth, *h*/mm	2	2	0.5
Spiral groove diameter, d_0_/mm	32	30	16
Spiral groove gap, *h*′/mm	0.15	0.13	0.10
Spiral rising angle, *φ*/degree	30	20	15

**Table 4:** The coding and level of the structural parameters of the bleed stage rotor.

Code	Parameters	Level		
		−1	0	1
K	Blade chord length, *b*/mm	3	4	5
M	Blade spacing, *a*/mm	4	5	6
N	Blade inclination angle, *α*/degree	20	30	40

**Table 5:** The design scheme of bleed stage rotor structural parameters and its pumping performance.

No.	K	M	N	Compression Ratio	Flow Rate (L/s)
1	3	4	30	2.42	8.69
2	5	4	30	2.46	8.37
3	3	6	30	2.21	9.35
4	5	6	30	2.38	8.97
5	3	5	20	2.87	7.56
6	5	5	20	3.18	7.34
7	3	5	40	2.26	10.37
8	5	5	40	2.22	9.88
9	4	4	20	3.23	7.33
10	4	6	20	3.08	7.77
11	4	4	40	2.36	9.97
12	4	6	40	2.08	10.78
13	4	5	30	2.51	9.47
14	4	5	30	2.56	9.54
15	4	5	30	2.54	9.46

**Table 6:** The coding and level of the structural parameters of the compression stage rotor.

Code	Parameter	Level		
		−1	0	1
A	Gap distance *h*′/mm	0.05	0.15	0.25
B	Spiral groove depth *h*/mm	0.5	2.5	4.5
C	Spiral rising angle *φ*	10	20	30

**Table 7:** The design scheme of compression stage rotor structural parameters and its pumping performance.

No.	A	B	C	Compression Ratio	Flow Rate (L/s)
1	0.05	0.5	20	60.46	1.15
2	0.25	0.5	20	26.18	1.23
3	0.05	4.5	20	111.76	2.57
4	0.25	4.5	20	82.72	2.45
5	0.05	2.5	10	163.57	1.45
6	0.25	2.5	10	116.71	1.42
7	0.05	2.5	30	105.28	2.58
8	0.25	2.5	30	58.75	2.77
9	0.15	0.5	10	74.03	0.92
10	0.15	4.5	10	137.28	1.67
11	0.15	0.5	30	38.75	1.28
12	0.15	4.5	30	47.31	2.91
13	0.15	2.5	20	164.56	2.39
14	0.15	2.5	20	167.64	2.35
15	0.15	2.5	20	171.49	2.31

**Table 8:** The structural parameters for the maximum single response target.

Single Response Target	Spiral Groove Gap *h*′/mm	Spiral Groove Gap *h*/mm	Spiral Rising Angle *φ*	Flow Rate *S*/(L/s)	Compression Ratio *C*
*C*_max_	0.163	3.321	12.895°	1.943	182.050
*S*_max_	0.05	4.5	30°	3.949	20.735

**Table 9:** The final design scheme for the compound molecular pump.

	Structural Parameters	Value	Compression Ratio C	Flow Rate S/(L/s)
Bleed stage rotor	Blade chord length, *b*/mm	4.629	2.49	10.16
	Blade spacing, *a*/mm	5.472		
	Blade inclination angle, *α*/degree	36.010		
Compression stage rotor	Gap distance, *h*′/mm	0.174	158.05	2.62
	Spiral groove depth, *h*/mm	3.288		
	Spiral rising angle, *φ*/degree	22.781		

**Table 10:** The pumping performance of the compound molecular pump before and after optimization.

	Compression Stage Rotor	Before Optimization	After Optimization	Improvement
Bleed stage rotor	Blade chord length, *b*/mm	4	4.7	-
	Blade spacing, *a*/mm	4	5.5	
	Blade inclination angle, *α*/degree	30	36	
	Compression ratio *C*	2.46	2.52	+2.4%
	Flow rate *S*/(L/s)	8.87	10.26	+15.6%
Compression stage rotor	Gap distance, *h*′/mm	0.15	0.17	-
	Spiral groove depth, *h*/mm	2	3.3	
	Spiral rising angle, *φ*/degree	30	23	
	Compression ratio *C*	102.29	156.23	+52.7%
	Flow rate *S*/(L/s)	2.58	2.74	+6.2%

## Data Availability

The raw data supporting the conclusions of this article will be made available by the authors on request.

## Nomenclature

*a*	Blade spacing (mm)
*b*	Blade chord length (mm)
*C*	Compression ratio
d_0_	Spiral groove diameter (mm)
*F_ce_*	centrifugal force (N)
*F_cr_*	Coriolis force (N)
*F_o_*	Euler force (N)
*F_t_*	the total force exerted on gas molecules (N)
*h*	Spiral groove depth (mm)
*h*′	Gap distance (mm)
*h*′	Spiral groove gap (mm)
*l*	Compression drum length (mm)
*m_p_*	molecular weight of the gas (kg)
*S*	Flow rate (L s^−1^)
*v*	velocity vector (m s^−1^)
*α*	Blade inclination angle (degree)
*γ*	Number of spiral groove
*φ*	Spiral rising angle (degree)
*θ*	the normal angle between the solid angle and area unit (degree)
*ω*	the rotational angular velocity of a rotating reference frame (rad s^−1^)
